# Light Absorption Analysis and Optimization of Ag@TiO_2_ Core-Shell Nanospheroid and Nanorod

**DOI:** 10.3390/nano14040325

**Published:** 2024-02-07

**Authors:** Dilishati Wumaier, Paerhatijiang Tuersun, Shuyuan Li, Yixuan Li, Meng Wang, Dibo Xu

**Affiliations:** Xinjiang Key Laboratory for Luminescence Minerals and Optical Functional Materials, School of Physics and Electronic Engineering, Xinjiang Normal University, Urumqi 830054, China; dr504871382@mail.ustc.edu.com (D.W.); lishuyuan@xjnu.edu.cn (S.L.); 18147053828@163.com (Y.L.); wangmeng091999@163.com (M.W.); xu13597881988@163.com (D.X.)

**Keywords:** core-shell nanoparticles, localized surface plasmon resonance, light absorption, finite element method, photothermal therapy

## Abstract

For photothermal therapy of cancer, it is necessary to find Ag @TiO_2_ core-shell nanoparticles that can freely tune the resonance wavelength within the near-infrared biological window. In this paper, the finite element method and the size-dependent refractive index of metal nanoparticles were used to theoretically investigate the effects of the core material, core length, core aspect ratio, shell thickness, refractive index of the surrounding medium, and the particle orientation on the light absorption properties of Ag@TiO_2_ core-shell nanospheroid and nanorod. The calculations show that the position and intensity of the light absorption resonance peaks can be freely tuned within the first and second biological windows by changing the above-mentioned parameters. Two laser wavelengths commonly used in photothermal therapy, 808 nm (first biological window) and 1064 nm (second biological window), were selected to optimize the core length and aspect ratio of Ag@TiO_2_ core-shell nanospheroid and nanorod. It was found that the optimized Ag@TiO_2_ core-shell nanospheroid has a stronger light absorption capacity at the laser wavelengths of 808 nm and 1064 nm. The optimized Ag@TiO_2_ core-shell nanoparticles can be used as ideal therapeutic agents in photothermal therapy.

## 1. Introduction

The treatment of cancer, a disease that poses a significant threat to human health, has consistently remained a pivotal research focus within the medical field [1]. Conventional cancer treatments mainly include surgery, chemotherapy, and radiation therapy. These conventional therapies, although partially effective in controlling cancer progression, present significant challenges for cancer treatment. They also have some drawbacks, such as unstable treatment effects and undesirable side effects [2]. Therefore, it is imperative to identify potent cancer treatment modalities.

Photothermal therapy (PTT) is an effective technique that uses photothermal conversion to treat cancer [3]. Nanomaterial-based PTT has been widely used in clinical treatment and scientific research [4,5]. In the process of PTT, a laser is used to irradiate the tumor site enriched with photothermal agents, which can convert the absorbed light energy into heat energy to ablate cancer cells. PTT is relatively simple to perform and has the advantages of a fast recovery, fewer complications, and shorter hospital stays. The laser wavelength is highly desired in the near-infrared (NIR) region (650–1350 nm). In the first NIR biological window (650–950 nm) and the second NIR biological window (1000–1350 nm), light absorption by biological tissues reaches the minimum, leading to maximum light penetration through tissue and minimizing autofluorescence [6,7,8,9]. Au nanoparticles (e.g., nanorods, nanoshells, nanostars, and nanocages) are selected as excellent photothermal agents in PTT because of efficient photothermal conversion, good stability, and biocompatibility [1,4,6,10]. However, Au nanoparticles are more expensive than most other metal nanoparticles. Therefore, it is necessary to develop photothermal agents with low cost and high photothermal conversion efficiency.

The availability of metal nanoparticles as therapeutic agents in PTT is attributed to their localized surface plasmon resonance (LSPR). When the LSPR is excited, metal nanoparticles can strongly absorb the incident light and convert it into heat [11,12]. Compared with Au nanoparticles, Ag nanoparticles are cheaper and have better LSPR properties [13]. However, the poor stability, easy oxidation, and toxicity of Ag nanoparticles limit their further applications in PTT. Therefore, the introduction of a protection layer outside of the Ag nanoparticles is needed. Recently, Ag nanoparticles were coated with a thin layer of oxides (such as SiO_2_, Cu_2_O, TiO_2_, Fe_3_O_4_, and ZnO) to form Ag@oxide core-shell nanoparticles [14,15,16,17,18,19,20,21,22,23,24,25]. In addition to protecting the Ag nanoparticles, the oxide layer can also redshift the LSPR wavelength of Ag nanoparticles. The high refractive index of TiO_2_ results in a larger redshift of LSPR wavelength toward the NIR biological window with an ultra-thin layer. The ultra-thin layer avoids heat loss and enhances the heat efficiency in the photothermal applications [19]. Various synthesis methods have been developed to prepare Ag@TiO_2_ core-shell nanoparticles in the last decades [26,27,28]. Most of the Ag@TiO_2_ core-shell nanoparticles are spherical or nearly spherical. The LSPR wavelength of the Ag@TiO_2_ core-shell nanospheres is within the visible light range, which cannot meet the need of PTT. Therefore, it is imperative to identify non-spherical Ag@TiO_2_ core-shell nanoparticles that offer the flexibility to precisely tune LSPR wavelengths within the NIR biological window. Nie et al. [19] successfully synthesized Ag@TiO_2_ core-shell nanoprisms that act as efficient photothermal agents for melanoma cancer treatment. They possess high photothermal conversion efficiency and strong light absorption at the laser wavelength of 808 nm. To the best of our knowledge, the application of other non-spherical Ag@TiO_2_ core-shell nanoparticles in PTT has not been reported. Moreover, a comprehensive numerical simulation of light absorption by non-spherical Ag@TiO_2_ core-shell nanoparticles has not been conducted. Therefore, the goal of this work is to fill this gap by providing theoretical analysis and optimization for the light absorption properties of Ag@TiO_2_ core-shell nanospheroids and nanorods.

## 2. Model and Method

The optical properties of non-spherical nanoparticles can be simulated using numerical methods, including finite element method (FEM), finite difference time domain (FDTD), discrete dipole approximation (DDA), and boundary element method (BEM) [29,30,31]. In this work, the light absorption properties of Ag@TiO_2_ core-shell nanoparticles were numerically simulated by using FEM with the size-dependent refractive index model. The main reasons for choosing the FEM are as follows: Firstly, FEM allows for easier modeling of complex geometrical and irregular shapes. Secondly, FEM can solve the problem with a high degree of accuracy. Finally, FEM can be used to model complex physical phenomena that involve multiple types of interactions, such as electromagnetic fields and photo-thermal coupling. These are also the main advantages of FEM.

### 2.1. Simulation Model

**Figure 1:**
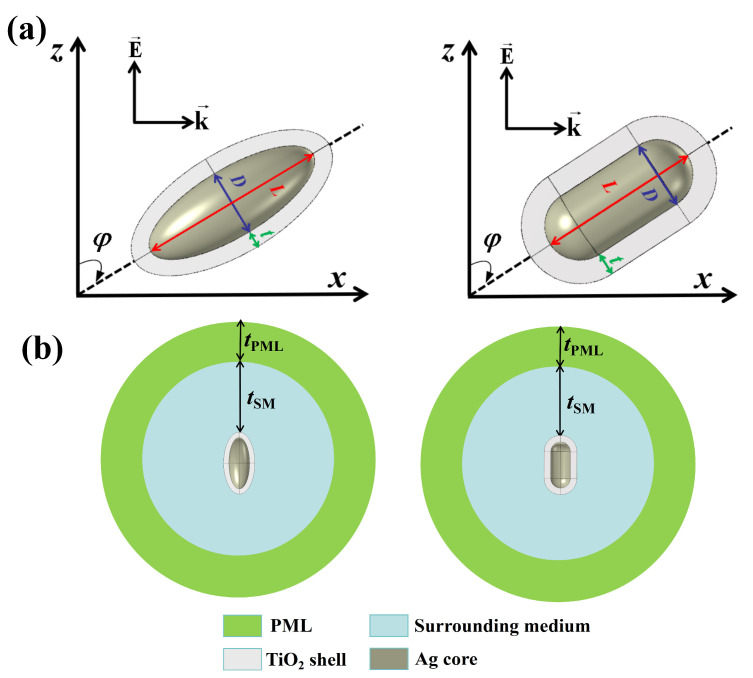
(**a**) Schematic of the geometry and orientation of Ag@TiO_2_ core-shell nanoparticle. (**b**) FEM simulation region.

Figure 1 shows the geometric model, orientation, and simulation region of the Ag@TiO_2_ core-shell nanospheroid and nanorod. As shown in Figure 1a, the Ag@TiO_2_ core-shell nanospheroid consists of a nanospheroid Ag core and a homogeneous TiO_2_ shell. The Ag@TiO_2_ core-shell nanorod consists of a rod-shaped Ag core and a homogeneous TiO_2_ shell. They can be represented by three geometric parameters: *L* for particle core length, *D* for particle core diameter, and *t* for particle shell thickness. In addition to the above three geometric parameters, the particle size can also be represented by the core aspect ratio *AR* = *L/D*. It should be noted that we considered a long spheroid (*L* > *D*) in this paper. In the simulation, it is assumed that the incident light propagates along the *x*-axis, the electric field component of the incident light oscillates along the *z*-axis, the rotation axis of the two particles is in the *xoz* plane, and *φ* represents the angle between the rotation axis and the *z*-axis (i.e., the azimuth). As shown in Figure 1b, a spherical region with a minimum thickness *t*_SM_ of 1/2 wavelength is taken as the surrounding medium (SM) layer outside of the particle. A spherical region with a thickness of 1/4 wavelength *t*_PML_ is taken as a perfectly matched layer (PML) outside of the SM layer. The SM layer defines the region where the light absorption and scattering parameters are calculated. The outer boundary of the SM layer is used as the scattering boundary. The PML is an artificial absorbing layer that truncates computational regions in numerical methods to simulate problems with open boundaries. The waves are completely absorbed in the PML to avoid them returning as reflections. In the calculations, the wavelength of incident light is varied from 400 nm to 1200 nm, so that the thicknesses of SM and PML are set as 1/2 and 1/4 wavelengths, respectively, instead of constant values. This helps to improve the computation accuracy at long wavelengths.

### 2.2. Calculation of Light Absorption

The light absorption capacity of an individual nanoparticle can be quantitatively described by the absorption cross-section *C*_abs_, which is defined as the ratio of the optical power absorbed by the particle to the incident light intensity. It can be expressed as follows [32]:

(1) $$C_{\mathrm{abs}}=\frac{1}{I_{i}}{∭}_{V_{p}}Q_{h}dV,$$

where *Q*_h_ represents the optical power density absorbed by the particle, *V*_p_ is the volume of the particle, and $I_{i}=(n_{m}E_{0}^{2})/(2Z_{0})$ is the intensity of the incident light, where *n*_m_ is the refractive index of the surrounding medium, *E*_0_ is the amplitude of the incident electric field (*E*_0_ = 1 in the simulation), and *Z*_0_ is the wave impedance of the vacuum.

The amount of material contained in nanoparticles of different sizes is different, resulting in different light absorption capacities. Therefore, to fairly compare the light absorption capacity of nanoparticles with different sizes, the ratio of the absorption cross-section *C*_abs_ to the volume *V*_p_ of the particles, *A*_abs_ = *C*_abs_/*V*_p_ (called the volume absorption coefficient), is used to investigate the light absorption properties of nanoparticles. When calculating the volume absorption coefficient *A*_abs_ of nanoparticles, it is necessary to obtain the refractive index of the nanoparticles. In the case of metal nanoparticles, the refractive index is not only related to the frequency of incident light but also to the size of the nanoparticles. This is because when the size of the particle is less than the mean free path of the free electrons, the collision between the free electrons and the surface of the particle is strengthened, and the surface scattering of free electrons cannot be ignored [33]. Under the circumstances, the size-dependent refractive index of metal nanoparticles can be expressed as follows [34]:

(2) $$n_{\mathrm{nano}\;}\left(\omega ,L_{eff}\right)=\sqrt{n_{\mathrm{bulk}\;}^{2}(\omega )+\frac{\omega_{p}^{2}}{\omega^{2}+i\omega v_{f}/l_{\infty }}-\frac{\omega_{p}^{2}}{\omega^{2}+i\omega \left(v_{f}/l_{\infty }+Av_{f}/L_{eff}\right)}},$$

where *ω* is the angular frequency of the incident light, *n*_bulk_ is the complex refractive index of bulk metals, *ω*_p_ is the plasma frequency, *v*_f_ is the Fermi velocity, *l*_∞_ is the mean free path of the free electrons, *A* is a dimensionless parameter close to 1 (1 in this paper), and *L*_eff_ is the effective free electron mean free path, which is usually taken as $L_{\mathrm{eff}}=R_{\mathrm{eff}}=\sqrt[{3}]{3V/4\pi }$. In this paper, we considered the core-shell nanoparticles with metal cores. For metals, i.e., Au, Ag, and Cu, *n*_bulk_ is obtained from the data published by Jonhson and Christy [35], and the values of plasma frequency *ω*_p_, Fermi velocity *v*_f_, and mean free path of the free electrons *l*_∞_ are obtained from published papers [36,37,38], as shown in Table 1. The refractive index of TiO_2_ is obtained from the data published by Sharma et al. [39].

### 2.3. Numerical Verification of FEM

**Figure 2:**
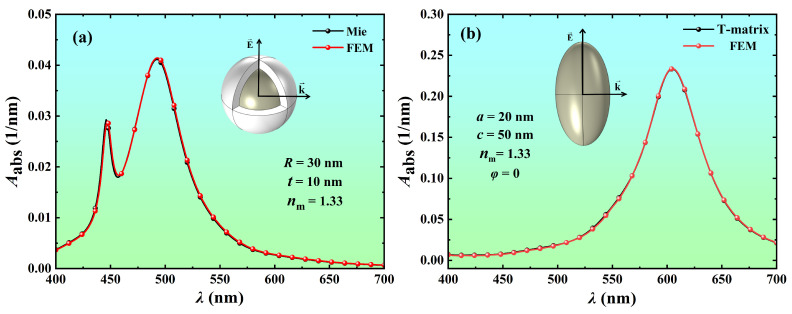
Absorption spectra of (**a**) Ag@TiO_2_ core-shell nanosphere and (**b**) Ag nanospheroid in water (*n*_m_ = 1.33). (**a**) The core radius *R* and shell thickness *t* of the core-shell nanosphere are 30 nm and 10 nm, respectively. (**b**) The minor semi-axis *a* and the major semi-axis *c* of the nanospheroid are 20 nm and 50 nm, respectively, and the azimuth *φ* is 0°.

To verify the accuracy and reliability of the FEM simulation results, we compared the FEM calculation results with the rigorous Mie theory [40] and the T-matrix method [41], as shown in Figure 2. Figure 2a shows the absorption spectra of Ag@TiO_2_ core-shell nanosphere with a core radius *R* of 30 nm and a shell thickness *t* of 10 nm in water (*n*_m_ = 1.33) obtained by Mie theory and FEM, respectively. Figure 2b shows the absorption spectra of Ag nanospheroid in water (*n*_m_ = 1.33) with a minor semi-axis *a* of 20 nm and a major semi-axis *c* of 50 nm obtained by T-matrix [42] and FEM, respectively. The calculation results show that the FEM simulation results agree well with the results calculated by the Mie theory and T-matrix method. It verifies that the FEM simulation is accurate and reliable.

## 3. Results and Discussion

The light absorption properties of nanoparticles are significantly affected by the material, shape, size, orientation, and the surrounding medium. To better understand the variation of the light absorption properties of nanoparticles with these influencing factors, two typical non-spherical core-shell structures, core-shell nanospheroid and nanorod, were selected as research objects to quantitatively analyze the influence of the core material, core length, core aspect ratio, shell thickness, the refractive index of the surrounding medium, and particle orientation on the light absorption properties. Moreover, the size parameters of nanoparticles were optimized for PTT applications. Except for the analysis of the effect of the environmental refractive index on the light absorption properties, the refractive index of the surrounding medium was taken as 1.44, which is the refractive index of the subcutaneous fat [43].

### 3.1. Effect of Core Material on Light Absorption

**Figure 3:**
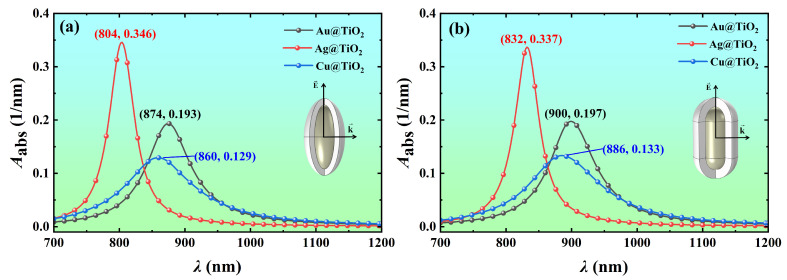
Variation of the volume absorption coefficient *A*_abs_ of Ag@TiO_2_, Au@TiO_2_, and Cu@TiO_2_ core-shell (**a**) nanospheroid and (**b**) nanorod with the wavelength *λ*. In the simulation, the core length *L* is 60 nm, the core aspect ratio *AR* is 3, the shell thickness *t* is 10 nm, the refractive index of the surrounding medium *n*_m_ is 1.44, and the azimuth *φ* is 0°.

To select the core material with high light absorption, the volume absorption coefficients of Ag@TiO_2_, Au@TiO_2_, and Cu@TiO_2_ core-shell nanospheroid and nanorod with the wavelength were calculated, as shown in Figure 3. It is found that the nanoparticles of Ag core have stronger light absorption at the resonance wavelength compared with the nanoparticles of Au and Cu cores. Ag@TiO_2_ nanospheroid has a larger volume absorption coefficient of 0.346 nm^−1^ at the resonance wavelength of 804 nm, which is 1.8 and 2.7 times that of Au@TiO_2_ and Cu@TiO_2_ nanospheroid at the resonance wavelength, respectively. The resonance wavelength of the core-shell nanospheroid is between 804 nm and 874 nm (Figure 3a), and the resonance wavelength of the core-shell nanorod is between 832 nm and 900 nm (Figure 3b). They are all within the NIR biological window. Compared with nanoparticles with Ag and Cu cores, the nanoparticles with a Au core have a larger resonance wavelength. The core-shell nanorod has a larger resonance wavelength than that of the core-shell nanospheroid of the same size. In conclusion, Ag@TiO_2_ core-shell nanoparticles have the best light absorption properties. This finding is in agreement with the results for pure Ag nanoparticles [13]. Therefore, in the following investigations, only the absorption spectra of Ag@TiO_2_ core-shell nanoparticles will be discussed as a function of particle size, environmental refractive index, and particle orientation.

### 3.2. Effect of Particle Size on Light Absorption

To reveal the variation of the light absorption properties of Ag@TiO_2_ core-shell nanoparticles with size, the variation of absorption spectra with the core length, core aspect ratio, and shell thickness of the nanoparticles are quantitatively analyzed in this section.

#### 3.2.1. Effect of Core Length

**Figure 4:**
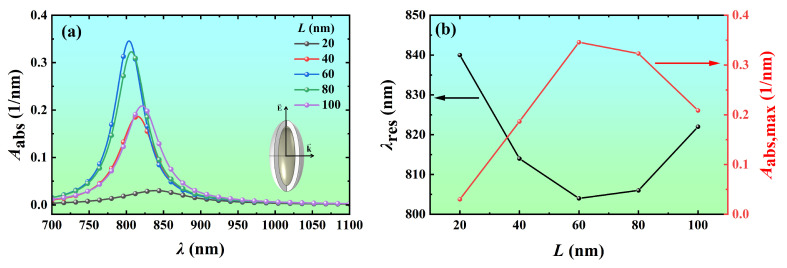

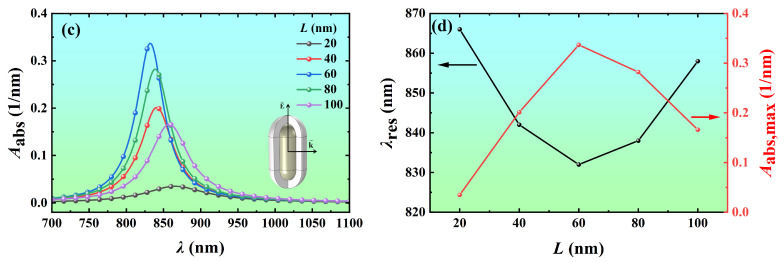
Variation of the absorption spectra and resonance peaks of Ag@TiO_2_ core-shell nanoparticles with core length *L*. The volume absorption coefficient *A*_abs_ of the Ag@TiO_2_ core-shell (**a**) nanospheroid and (**c**) nanorod of different core lengths as a function of the wavelength *λ*. The resonance wavelength and peak values of the Ag@TiO_2_ core-shell (**b**) nanospheroid and (**d**) nanorod as a function of *L*. In the simulation, the core aspect ratio *AR* is 3, the shell thickness *t* is 10 nm, the refractive index of the surrounding medium *n*_m_ is 1.44, and the azimuth *φ* is 0°.

To quantitatively analyze the effect of the core length on the absorption spectra, the volume absorption coefficients of Ag@TiO_2_ core-shell nanospheroid and nanorod with core lengths of 20 nm, 40 nm, 60 nm, 80 nm, and 100 nm were calculated as a function of wavelength, as shown in Figure 4. It can be seen that the resonance wavelength of the Ag@TiO_2_ core-shell nanorod is greater than that of the Ag@TiO_2_ core-shell nanospheroid with the same size. As the core length increases from 20 nm to 100 nm, the resonance wavelength of the Ag@TiO_2_ core-shell nanospheroid blueshifts from 840 nm to 804 nm and then redshifts to 822 nm, and the volume absorption coefficient at the resonance wavelength increases from 0.030 nm^−1^ to 0.346 nm^−1^ and then decreases to 0.209 nm^−1^ (Figure 4a,b). The resonance wavelength of the Ag@TiO_2_ core-shell nanorod blueshifts from 866 nm to 832 nm and then redshifts to 858 nm, and the volume absorption coefficient at the resonance wavelength increases from 0.035 nm^−1^ to 0.337 nm^−1^ and then decreases to 0.166 nm^−1^ (Figure 4c,d). The light absorption capacity of the nanoparticles at the resonance wavelength increases first and then decreases with the increase of core length. It is caused by the change in optical properties and electronic structure of the nanoparticles. Firstly, the LSPR frequency of nanoparticles will change with the increase in size, resulting in a change in light absorption. Secondly, the electronic density of states of nanoparticles will change with the increase in size, thus affecting their ability to absorb photons [13].

#### 3.2.2. Effect of Core Aspect Ratio

**Figure 5:**
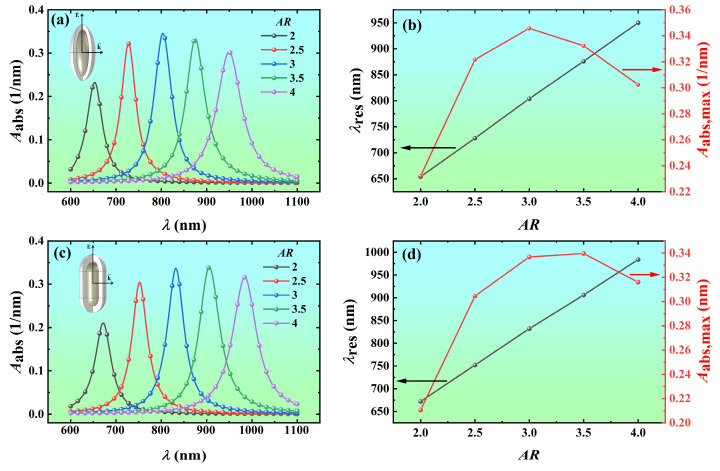
Variation of the absorption spectra and resonance peaks of Ag@TiO_2_ shell nanoparticles with the core aspect ratio *AR*. The volume absorption coefficient *A*_abs_ of Ag@TiO_2_ core-shell (**a**) nanospheroid and (**c**) nanorod of different core aspect ratios as a function of the wavelength *λ*. The resonance wavelength and peak values of the Ag@TiO_2_ core-shell (**b**) nanospheroid and (**d**) nanorod as a function of *AR*. In the simulation, the core length *L* is 60 nm, the shell thickness *t* is 10 nm, the refractive index of the surrounding medium *n*_m_ is 1.44, and the azimuth *φ* is 0°.

To quantitatively analyze the effect of the core aspect ratio on the absorption spectra, the volume absorption coefficients of the Ag@TiO_2_ core-shell nanospheroid and nanorod with core aspect ratios of 2, 2.5, 3, 3.5, and 4 were calculated as a function of wavelength, as shown in Figure 5. As the core aspect ratio increases from 2 to 4, the resonance wavelength of the Ag@TiO_2_ core-shell nanospheroid redshifts from 654 nm to 950 nm, and the volume absorption coefficient at the resonance wavelength increases from 0.232 nm^−1^ to 0.346 nm^−1^ and then decreases to 0.302 nm^−1^ (Figure 5a,b). The resonance wavelength of the Ag@TiO_2_ core-shell nanorod redshifts from 672 nm to 984 nm, and the volume absorption coefficient at the resonance wavelength increases from 0.211 nm^−1^ to 0.340 nm^−1^ and then decreases to 0.316 nm^−1^ (Figure 5c,d). The resonance wavelengths of the nanoparticles are significantly redshifted because the natural oscillation frequency of free electrons in Ag nanoparticles decreases with the increase of the core aspect ratio, resulting in a decrease in the resonance frequency (i.e., an increase in the resonance wavelength) [44]. Similar results for Au and Ag nanorods have been obtained during the last two decades [45,46,47,48,49].

#### 3.2.3. Effect of Shell Thickness

**Figure 6:**
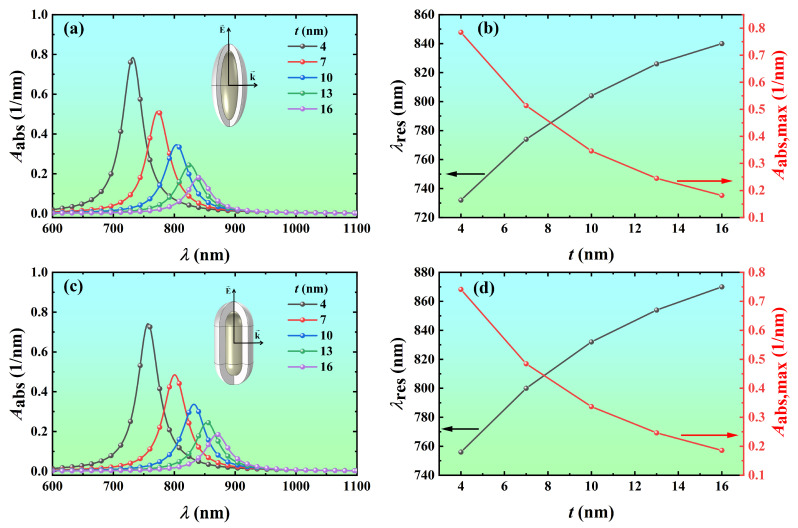
Variation of the absorption spectra and resonance peaks of Ag@TiO_2_ core-shell nanoparticles with shell thickness *t*. The volume absorption coefficient *A*_abs_ of the Ag@TiO_2_ core-shell (**a**) nanospheroid and (**c**) nanorod of different shell thickness as a function of the wavelength *λ*. The resonance wavelength and peak values of the Ag@TiO_2_ core-shell (**b**) nanospheroid and (**d**) nanorod as a function of *t*. In the simulation, the core length *L* is 60 nm, the core aspect ratio *AR* is 3, the refractive index of the surrounding medium *n*_m_ is 1.44, and the azimuth *φ* is 0°.

To quantitatively analyze the effect of the shell thickness on the absorption, the volume absorption coefficients of Ag@TiO_2_ core-shell nanospheroid and nanorod with shell thicknesses of 4 nm, 7 nm, 10 nm, 13 nm, and 16 nm were calculated as a function of wavelength, as shown in Figure 6. As the shell thickness increases from 4 nm to 16 nm, the resonance wavelength of the Ag@TiO_2_ core-shell nanospheroid redshifts from 732 nm to 840 nm, and the volume absorption coefficient at the resonance wavelength decreases from 0.785 nm^−1^ to 0.182 nm^−1^ (Figure 6a,b). The resonance wavelength of the Ag@TiO_2_ core-shell nanorod redshifts from 756 nm to 870 nm, and the volume absorption coefficient at the resonance wavelength decreases from 0.741 nm^−1^ to 0.186 nm^−1^ (Figure 6c,d).

In conclusion, by changing the core length, core aspect ratio, and shell thickness of the Ag@TiO_2_ core-shell nanoparticles, the resonance wavelength of the nanoparticles can be accurately adjusted in the visible to near-infrared wavelength range to meet the needs of practical applications.

### 3.3. Effect of Refractive Index of Surrounding Medium on Light Absorption

**Figure 7:**
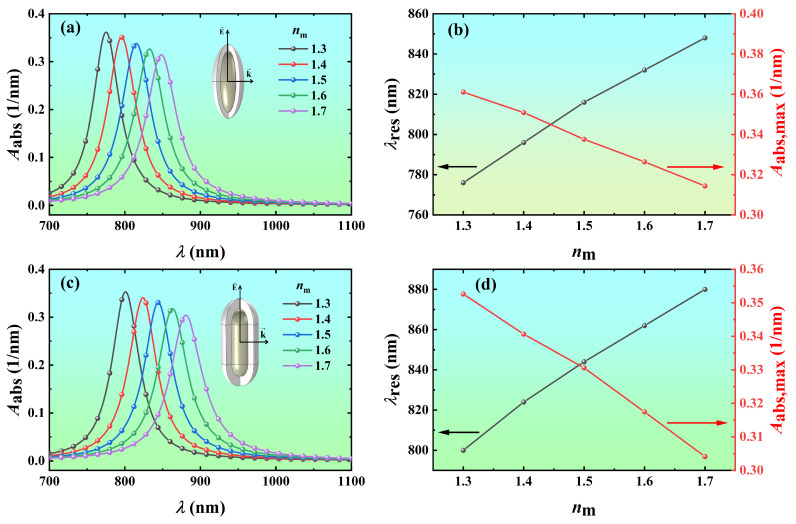
Variation of the absorption spectra and resonance peaks of Ag@TiO_2_ core-shell nanoparticles with the refractive index of the surrounding medium *n*_m_. The volume absorption coefficient *A*_abs_ of the Ag@TiO_2_ core-shell (**a**) nanospheroid and (**c**) nanorod in the different medium as a function of the wavelength *λ*. The resonance wavelength and peak values of the Ag@TiO_2_ core-shell (**b**) nanospheroid and (**d**) nanorod as a function of *n*_m_. In the simulation, the core length *L* is 60 nm, the core aspect ratio *AR* is 3, the shell thickness *t* is 10 nm, and the azimuth *φ* is 0°.

To further reveal the variation of light absorption properties of Ag@TiO_2_ core-shell nanoparticles with the refractive index of the surrounding medium, the volume absorption coefficients of Ag@TiO_2_ core-shell nanospheroid and nanorod in the different biological tissues (*n*_m_ = 1.3, 1.4, 1.5, 1.6, and 1.7) were calculated as a function of wavelength, as shown in Figure 7. As the refractive index of the surrounding medium increases from 1.3 to 1.7, the resonance wavelength of the Ag@TiO_2_ core-shell nanospheroid redshifts from 776 nm to 848 nm, and the corresponding volume absorption coefficient decreases from 0.361 nm^−1^ to 0.314 nm^−1^ (Figure 7a,b). The resonance wavelength of the Ag@TiO_2_ core-shell nanorod redshifts from 800 nm to 880 nm, and the corresponding volume absorption coefficient decreases from 0.353 nm^−1^ to 0.304 nm^−1^ (Figure 7c,d). The main reason for this phenomenon is that when the refractive index of the surrounding medium increases, the propagation speed of light slows down, so that the wavelength of light in the surrounding medium becomes shorter, increasing the wavelength of the incident light that excites the LSPR of nanoparticles. Overall, the LSPR of metal nanoparticles is highly sensitive to the refractive index of the surrounding medium. Therefore, nanoscale biological and chemical sensors have been developed using the shift of LSPR in response to changes in the local dielectric environment of the metal nanoparticles [50].

### 3.4. Effect of Particle Orientation on Light Absorption

**Figure 8:**
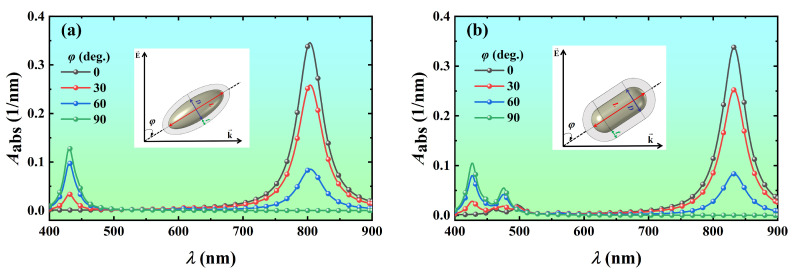
Variation of the volume absorption coefficient *A*_abs_ of Ag@TiO_2_ core-shell (**a**) nanospheroid and (**b**) nanorod of different orientations with the wavelength *λ*. In the simulation, the core length *L* is 60 nm, the core aspect ratio *AR* is 3, the shell thickness *t* is 10 nm, and the refractive index of the surrounding medium *n*_m_ is 1.44.

To investigate the variation of the light absorption of the Ag@TiO_2_ core-shell nanoparticles with the orientation of the particles, the volume absorption coefficients of the Ag@TiO_2_ core-shell nanospheroid and nanorod with four different orientations (*φ* = 0°, 30°, 60°, and 90°) were calculated as a function of the wavelength, as shown in Figure 8. It is found that two resonance peaks appeared in the absorption spectrum of the Ag@TiO_2_ core-shell nanoparticles. They correspond to the vibrations of free electrons along the axial direction of the particle (longitudinal LSPR, L-LSPR) and are perpendicular to the axial direction (transverse LSPR, T-LSPR). The charge accumulation in L-LSPR mode is relatively small, while that in T-LSPR mode is greater. The oscillation restoring force of the charge is proportional to the charge accumulation. Therefore, the electronic vibration frequency of L-LSPR is slower (i.e., longer resonance wavelength), while the electron vibration in T-LSPR resonance mode is faster (i.e., shorter resonance wavelength) [13]. When the azimuth is 0°, only the L-LSPR mode appears, the volume absorption coefficient of the Ag@TiO_2_ core-shell nanospheroid at the resonance wavelength of 804 nm is 0.346 nm^−1^ (Figure 8a), and the volume absorption coefficient of the Ag@TiO_2_ core-shell nanorod at the resonance wavelength of 832 nm is 0.338 nm^−1^ (Figure 8b). When the azimuth is 90°, only the T-LSPR mode appears, the volume absorption coefficient of the Ag@TiO_2_ core-shell nanospheroid at the resonance wavelength of 430 nm is 0.129 nm^−1^ (Figure 8a), and the volume absorption coefficient of the Ag@TiO_2_ core-shell nanorod at the resonance wavelength of 426 nm is 0.104 nm^−1^ (Figure 8b). When the azimuths are 30° and 60°, the L-LSPR and T-LSPR modes occur simultaneously, and the position of the resonance peaks is almost constant because the size and charge density of the nanoparticles do not change. No matter how the size parameters are changed, the T-LSPR wavelength is always in a very narrow visible range, while the L-LSPR wavelength can be tuned arbitrarily in the visible range up to NIR. Moreover, the L-LSPR intensity is much greater than the T-LSPR intensity. The L-LSPR mode makes Au nanorods have many potential applications in sensing, surface-enhanced spectroscopies, optoelectronics, photocatalysis, and biotechnologies [51,52,53]. Currently, there are various techniques for controlling the orientation of nanorods [54,55,56,57,58,59,60]. If the azimuth of all Ag@TiO_2_ core-shell nanoparticles is fixed at 0° by using these techniques, the light absorption capacity of Ag@TiO_2_ core-shell nanoparticles will be greatly enhanced.

### 3.5. Optimization of Light Absorption Properties

**Figure 9:**
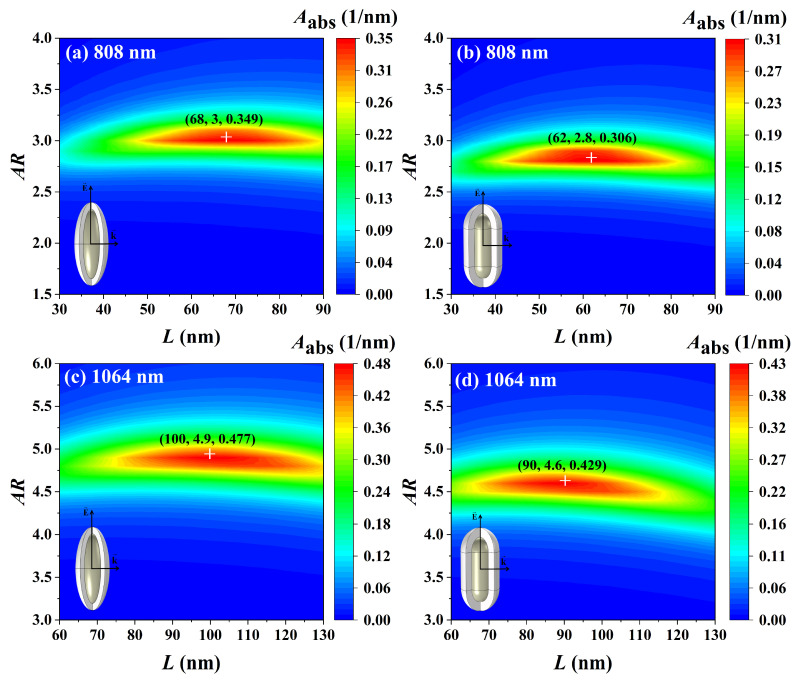
Optimization of size parameters of Ag@TiO_2_ core-shell (**a**,**c**) nanospheroid and (**b**,**d**) nanorod at the laser wavelengths of 808 nm and 1064 nm. In the simulation, the shell thickness *t* is 10 nm, the refractive index of biological tissue is 1.44, and the azimuth *φ* is 0°.

For the application of Ag@TiO_2_ core-shell nanoparticles in PTT, it is necessary to find optimal size parameters of Ag@TiO_2_ core-shell nanoparticles with the best light absorption properties. Different wavelengths of light have different penetration depths in biological tissues, and the degree of thermal damage to normal cells is also different. Therefore, according to the different treatment needs, choosing the proper light wavelength can better achieve the targeted and effective treatment. The incident light of 808 nm wavelength has relatively little thermal damage to healthy cells, but its penetration in biological tissues is limited. Therefore, it is only suitable for the treatment of superficial cancer cells. However, the incident light of 1064 nm wavelength has good penetration in biological tissues, and the thermal damage to healthy cells is relatively strong [6]. To meet the needs of different types of PTT, the two most commonly used lasers with wavelengths of 808 nm and 1064 nm were selected as light sources. The maximum volume absorption coefficients of the Ag@TiO_2_ core-shell nanoparticles and the corresponding optimal core length and aspect ratio were calculated, as shown in Figure 9. It is seen that, when the laser wavelength is 808 nm, the Ag@TiO_2_ core-shell nanospheroid with a core length of 68 nm and an aspect ratio of 3 has a maximum volume absorption coefficient of 0349 nm^−1^ (Figure 9a), and the Ag@TiO_2_ core-shell nanorod with a core length of 62 nm and an aspect ratio of 2.8 has a maximum volume absorption coefficient of 0.306 nm^−1^ (Figure 9b). The optimized Ag@TiO_2_ core-shell nanospheroid has a better light absorption capacity than the core-shell nanorod at 808 nm. When the laser wavelength is 1064 nm, the optimal size parameters and the maximum volume absorption coefficient both become larger (Figure 9c,d). In conclusion, the Ag@TiO_2_ core-shell nanospheroid has a larger light absorption capacity at the wavelengths of 808 nm and 1064 nm. The optimized Ag@TiO_2_ core-shell nanoparticles can be used as ideal photothermal conversion particles in the laser-based PTT.

## 4. Conclusions

In this paper, we investigated the variation of the light absorption properties of Ag@TiO_2_ core-shell nanospheroid and nanorod with the core material, size parameters, environmental refractive index, and particle orientation. We also obtained the optimal core length and aspect ratio of nanoparticles at the laser wavelengths of 808 nm and 1064 nm, which are commonly used in PTT. It was found that the core-shell nanoparticles with a Ag core have the best light absorption compared with the core-shell nanoparticles with Au and Cu cores. By changing the nanoparticle size parameters (core length, core aspect ratio, and shell thickness), the light absorption peaks of the Ag@TiO_2_ core-shell nanoparticles can be arbitrarily tuned in the visible range up to NIR. With the increase of the refractive index of the surrounding medium, the absorption resonance peak of the Ag@TiO_2_ core-shell nanoparticles redshifts, and the peak intensity gradually decreases. The change of particle orientation does not cause the shift of the absorption resonance peak of the Ag@TiO_2_ core-shell nanoparticles but can control the emergence of L-LSPR and T-LSPR modes. When the laser wavelength is 808 nm, the optimized Ag@TiO_2_ core-shell nanospheroid has a better light absorption capacity than the Ag@TiO_2_ core-shell nanorod. The Ag@TiO_2_ core-shell nanospheroid with a core length of 68 nm and an aspect ratio of 3 has a maximum volume absorption coefficient of 0349 nm^−1^. When the laser wavelength is 1064 nm, the optimal size parameters and the maximum volume absorption coefficient become larger. Optimized Ag@TiO_2_ core-shell nanoparticles can be used as ideal therapeutic agents in PTT.

## Figures and Tables

**Table 1:** The values of plasma frequency *ω*_p_, Fermi rate *v*_f_, and mean free path of the free electrons *l*_∞_ for Au, Ag, and Cu [36,37,38].

Metals	$\hbar \omega_{p}$ (eV)	*v*_f_ (m/s)	*l*_∞_ (nm)
Au	9.03	1.40 × 10^6^	42
Ag	9.01	1.39 × 10^6^	52
Cu	10.83	1.57 × 10^6^	45

## Data Availability

The original contributions presented in this study are included in the article; further inquiries can be directed to the corresponding authors.
